# A Novel Peptide-Based Enzyme-Linked Immunosorbent Assay (ELISA) for Detection of Neutralizing Antibodies Against NADC30-like PRRSV GP5 Protein

**DOI:** 10.3390/ijms26062619

**Published:** 2025-03-14

**Authors:** Shaohua Sun, Kaili Zhang, Jiajia Zhang, Pingping Zhang, Ping He, Dafu Deng, Sen Jiang, Wanglong Zheng, Nanhua Chen, Jianfa Bai, Jianzhong Zhu

**Affiliations:** 1 College of Veterinary Medicine, Yangzhou University, Yangzhou 225009, China; 2 Joint International Research Laboratory of Agriculture and Agri-Product Safety, Yangzhou University, Yangzhou 225009, China; 3 Comparative Medicine Research Institute, Yangzhou University, Yangzhou 225009, China; 4 Jiangsu Co-Innovation Center for Prevention and Control of Important Animal Infectious Diseases and Zoonoses, Yangzhou University, Yangzhou 225009, China; 5 Kansas State Veterinary Diagnostic Laboratory, Kansas State University, Manhattan, KS 66506, USA

**Keywords:** NADC30-like PRRSV, GP5 protein, epitope, peptide-based ELISA, neutralizing antibody

## Abstract

Porcine reproductive and respiratory syndrome (PRRS) is a pig respiratory disease threating the global swine industry. To combat PRRS, it is necessary of the effective diagnostic detection of antibody, including developing a neutralizing antibody against porcine reproductive and respiratory syndrome virus (PRRSV), especially the currently prevalent NADC30-like PRRSV in China. In this study, we prepared three monoclonal antibodies (mAbs) against NADC30-like PRRSV glycoprotein 5 (GP5) protein, and identified two corresponding precise epitopes (^155^WR^156^ and ^196^QWGRP^200^). In the neutralization test, ^196^QWGRP^200^ recognizing GP5 mAbs (11E6 and 12D1) exhibited obvious neutralizing activity, whereas the ^155^WR^156^ recognizing mAb (3A8) showed low neutralizing activity. Based on the two antigenic peptides, a peptide-based Enzyme-Linked Immunosorbent Assay (ELISA) was developed to detect antibodies against PRRSV, presenting high specificity, sensitivity, and repeatability. The concordance rate of the peptide-based ELISA and commercial IDEXX PRRSV X3 Ab ELISA in detection of 81 clinical samples was 82.7%. In conclusion, the GP5 peptide-based ELISA can be used for the detection of neutralizing antibodies against NADC30-like PRRSV, providing a rapid and reliable method for monitoring PRRSV infection.

## 1. Introduction

Porcine reproductive and respiratory syndrome (PRRS) is one of the most important infectious viral diseases in pig herds, which is caused by the porcine reproductive and respiratory syndrome virus (PRRSV) and characterized by reproductive disorders in sows and respiratory distress in piglets [1]. PRRSV can be divided into two species, the *Betaarterivirus suid 1* (PRRSV-1, European-type PRRSV, prototype Lelystad strain) and the *Betaarterivirus suid 2* (PRRSV-2, North American-type PRRSV, prototype VR-2332 strain) [2]. PRRSV-2 has often been regarded as the epidemiologically predominant species in China [3]. In 2006, the highly pathogenic PRRSV-2 (HP-PRRSV), characterized by high mortality, fever, and abortion rates, first appeared in southern China and devastated the Chinese swine industry [4]. In recent years, NADC30-like PRRSV-2 and NADC34-like PRRSV-2, which show much less pathogenicity against pigs than HP-PRRSV, have been circulating in China and become the dominant strains in field [5].

PRRSV is an enveloped, single-stranded, positive-sense RNA virus, which belongs to the genus *Betaarterivirus*, family *Arteriviridae*, and order *Nidovirales* [6]. The genome, approximately 15 kb in length, contains at least 10 known open reading frames (*ORFs*) [7]. *ORF1a* and *ORF1b* encode two polyproteins, pp1a and pp1ab, which are processed into at least 16 mature nonstructural proteins (NSPs) by viral proteases that play a major role in virus replication [8]. The remaining *ORFs* (*ORF2–7*) encode structural proteins including GP2a, GP2b, GP3–GP5, GP5a, M, and N proteins [8]. GP5 protein, a glycosylated envelope protein, is encoded by the PRRSV *ORF5* gene, with the molecular weight of approximately 25 kD [9]. As the most mutable structural protein, GP5 is frequently used for phylogenetic analyses of PRRSV [10,11]. Among different viral structural proteins, GP5 is the major virulence determinant and plays an important role in virus infection [12,13]. Moreover, GP5 contains important immune domains associated with virus neutralization, serving as a good target antigen for developing new vaccines [9,14].

The effective diagnosis of PRRS is important to control and prevention [15]. The detection of antibodies, including the neutralizing antibody against PRRSV, not only promotes diagnosis of PRRS, but also aids in assessing the immune protection status of pig herds [15,16]. Currently, several methods have been applied for the detection of PRRS antibodies, including immunoperoxidase monolayer assays, indirect fluorescent assays, serum neutralization tests, and enzyme-linked immunosorbent assays (ELISAs) [15]. The immunoperoxidase monolayer assays and indirect fluorescent assays are subjective, laborious, and expensive and are mainly suitable for laboratory testing [15]. Serum neutralization tests can examine neutralizing antibodies produced by PRRSV infection in pigs; nevertheless, it is unsuitable for diagnosing acute infection and is also laborious [17]. ELISAs are a popular method for the detection of PRRSV antibodies because of their high specificity, high sensitivity, and low cost [15]. Several indirect ELISAs detecting PRRSV GP5 antibodies have been established [18,19,20]; however, in these indirect ELISAs, the recombinant expression vector was firstly constructed. Subsequently, the protein was expressed and purified, and then used as the coating antigen. Thus, the antigen preparation for the ELISAs takes a long time and lacks a precision.

In the current study, we generated three monoclonal antibodies (mAbs) specific for NADC30-like PRRSV GP5 and identified two precise antigenic epitopes that mediate virus neutralization. Based on the two antigenic epitopes, a peptide-based ELISA was developed to detect neutralizing antibodies against NADC30-like PRRSV.

## 2. Results

### 2.1. Expression and Purification of the Recombinant NADC30-like PRRSV GP5 Truncated Protein

**Figure 1:**
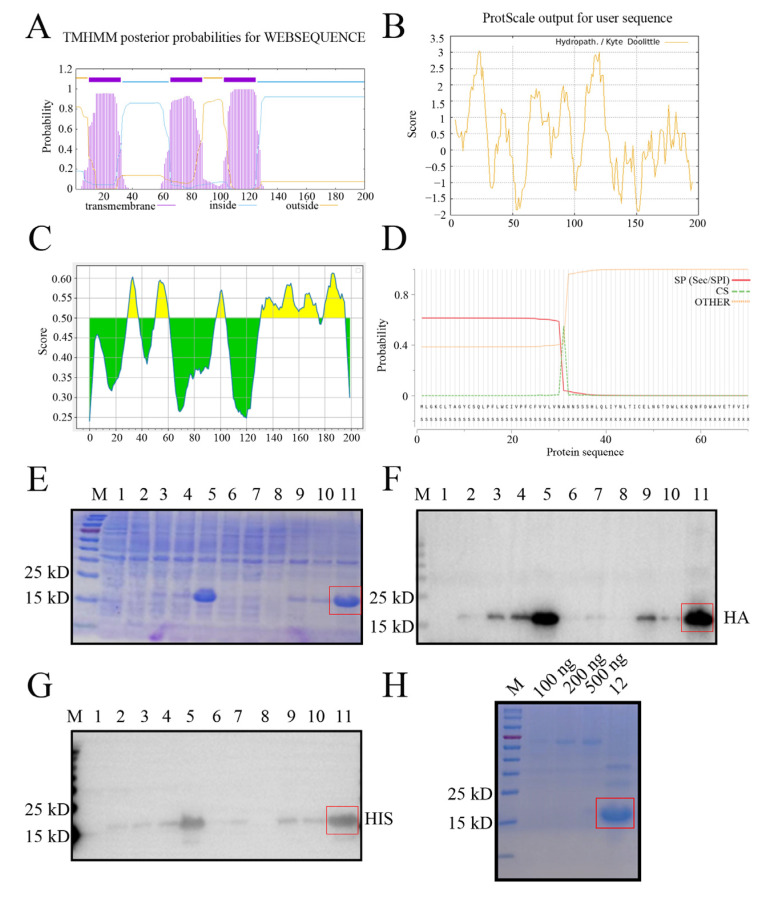
Expression and purification of the recombinant PRRSV GP5 truncation protein. (**A**–**C**) Analysis of the PRRSV GP5 protein transmembrane regions (**A**), hydrophilicity (**B**), and B cell antigenic epitopes colored in yellow (**C**) using online tools, as described in the Section 4. The higher the score, the higher probability in (**A**–**C**). (**D**) The signal peptide was predicted by the SignalP-5.0. SP: signal peptide. CS: cleavage site position. OTHER: no signal peptide at all. (**E**–**G**) The bacterial lysate proteins were detected by SDS-PAGE (**E**) and by Western blotting with anti-HA mAb (**F**) and anti-His mAb (**G**). (**H**) The purified GP5 protein was verified by SDS-PAGE against different amounts of BSA as the controls. Lane 1: pDEST527 vector transformed BL21; lane 2: pDEST527-GP5 transformed BL21; lanes 3, 6, 9: pDEST527-GP5 transformed BL21 induced by IPTG at 16 °C for 12 h; lanes 4, 7, 10: pDEST527-N transformed BL21 induced by IPTG at 16 °C for 24 h; and lanes 5, 8, 11: pDEST527-N transformed BL21 induced by IPTG at 37 °C for 12 h. Lanes 1–5: whole bacterial lysates; lanes 6–8, the supernatants of lysed bacteria; lanes 9–11, the precipitates of lysed bacteria. Lane 12: purified recombinant GP5 protein. M: protein marker. The greater expressed GP5 in the inclusion body and the purified GP5 are marked by a red box.

Bioinformatics analysis revealed that the GP5 protein of NADC30-like porcine reproductive and respiratory syndrome virus (PRRSV) SD17-38 strain is a multiple transmembrane protein, with the transmembrane (TM) regions located at 10–32, 66–88, and 103–125 amino acids (aa) (Figure 1A), which exhibit high hydrophobicity (Figure 1B). Both the extramembrane region (89–102 aa) and intramembrane regions (33–65, 126–200 aa) contain the B cell antigenic epitopes highlighted in yellow color (Figure 1C). The signal peptide was predicted to be 1–31 aa, with an accurate cleavage site between residues 31 and 32 (Figure 1D). Given that the intramembrane regions (33–65, 126–200 aa) exhibited higher hydrophilicity (Figure 1B) and contain more antigenicity (Figure 1C), the recombinant prokaryotic GP5 plasmid expressing the two fused intramembrane regions (33–65, 126–200 aa) was constructed and the plasmid transformed into *E. coli* cells was induced by IPTG to express the GP5 fused truncation protein. The recombinant NADC30-like PRRSV GP5 protein was determined to mainly express in the form of an inclusion body at 37 °C for 12 h by sodium dodecyl sulphate-polyacrylamide gel electrophoresis (SDS-PAGE) (Figure 1E). The expressed GP5 protein, with an expected molecular weight of 15–25 kD, was confirmed in Western blotting (WB) by both anti-HA mAb (Figure 1F) and anti-His mAb (Figure 1G). Further, the purified GP5 protein exhibited a high purity and amount in SDS-PAGE (Figure 1H). The data indicated that the purified recombinant GP5 truncation protein was suitable for immunization.

### 2.2. Preparation and Identification of GP5 Protein Specific mAbs

**Figure 2:**
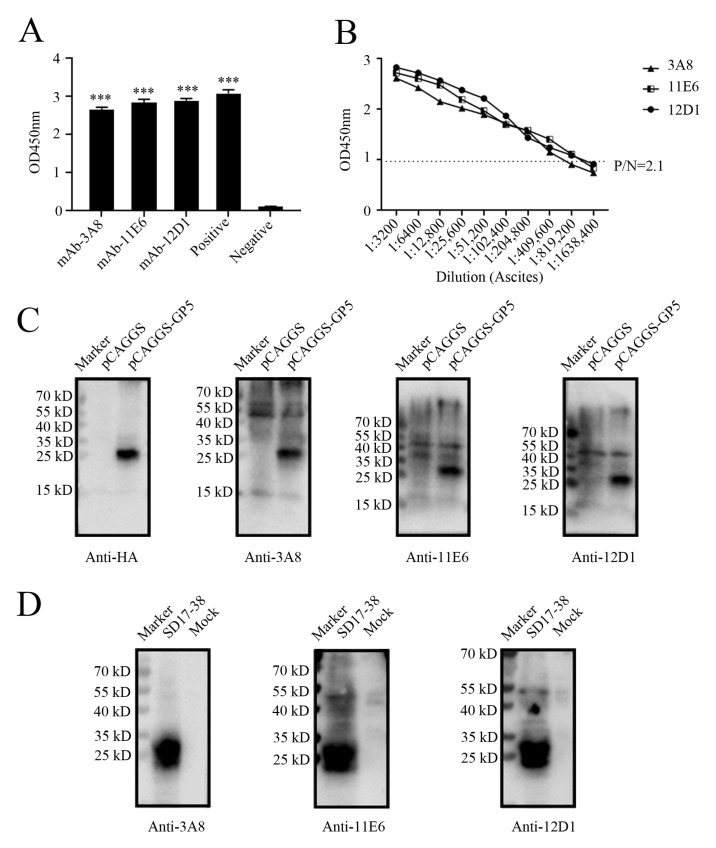
Identification of GP5 specific mAbs by Western blotting (WB). (**A**) Detection of GP5 antibodies in supernatants from the hybridoma cell clones (3A8, 11E6, and 12D1) by indirect ELISA. Serum from immunized mice was used as the positive control and SP2/0 cell supernatant was used as the negative control. ***: *p* < 0.001 vs. negative control. (**B**) Determination of the titers of ascites mAbs (3A8, 11E6, and 12D1) through a GP5 indirect ELISA. The ascites fluids were subjected to 2-fold serial dilutions, and normal mouse serum was used as negative control. (**C**,**D**) WB was used to confirm the reactivity of GP5 mAbs (3A8, 11E6, and 12D1) with GP5 in HEK293T cells transfected with pCAGGS-GP5-HA or the pCAGGS-HA vector control (**C**), and to verify the reactivity of GP5 in 3D4/21-CD163 cells mock infected or infected with 0.1 MOI SD17-38 (**D**).

**Figure 3:**
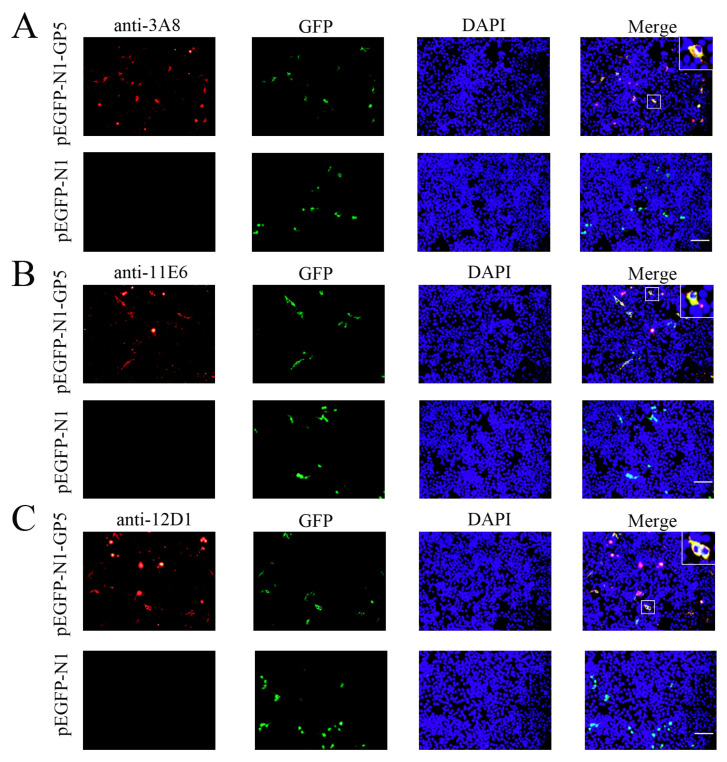
The specific reactivity of GP5 mAbs with the ectopic GP5 in IFA. HEK293T cells were transfected with the pEGFP-N1-GP5 or pEGFP-N1 vector for 24 h. After fixation, cells were stained with GP5 mAbs 3A8 (**A**), 11E6 (**B**), and 12D1 (**C**), together with the anti-mouse IgG (H + L) secondary antibody DyLight™ 594 (red). Cell nuclei were counterstained with DAPI (blue). The boxed areas were magnified and placed in the upper-right corners of the merged images, clearly illustrating that the GP5 protein is located in the cytoplasm. Scar bar: 100 μm.

**Figure 4:**
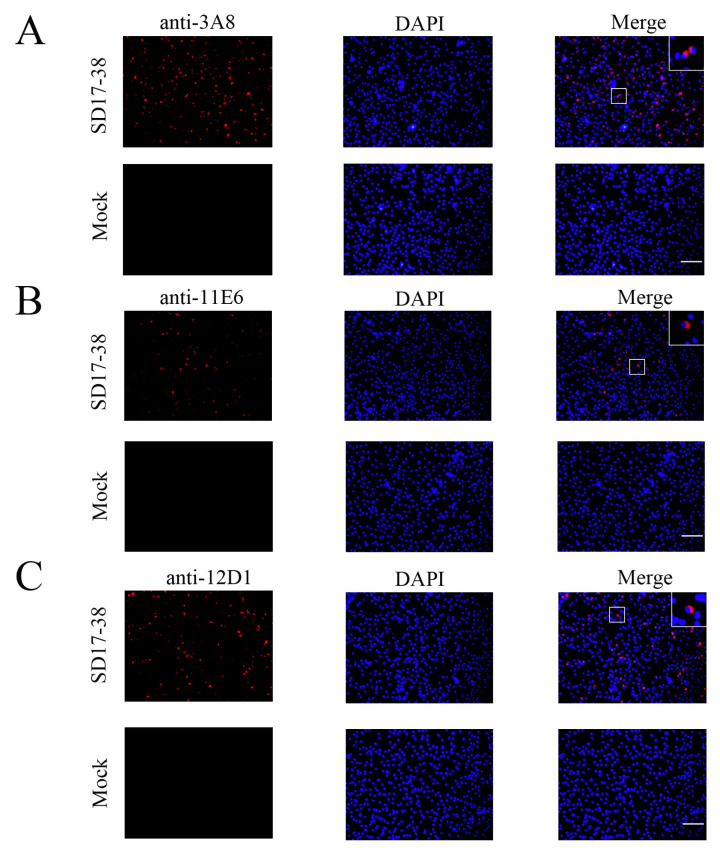
The specific reactivity of GP5 mAbs with PRRSV expressed GP5 in IFA. 3D4/21-CD163 cells were mock infected or infected with 0.1 MOI PRRSV-2 SD17-38 for 72 h. After fixation, the cells were stained with GP5 mAbs 3A8 (**A**), 11E6 (**B**), and 12D1 (**C**), together with the anti-mouse IgG (H + L) secondary antibody DyLight™ 594 (red). Cell nuclei were counterstained with DAPI (blue). The boxed areas were magnified and placed in the upper-right corners of the merged images, clearly illustrating that GP5 protein is located in the cytoplasm. Scar bar: 100 μm.

Via cell fusion, we obtained three hybridoma cell clones (3A8, 11E6, and 12D1) with notably high positive readings after screening and subcloning thrice in a GP5 coated indirect enzyme-linked immunosorbent assay (ELISA) (Figure 2A). The ascites titers of hybridoma cell clones 3A8, 11E6, and 12D1 were measured using the indirect ELISA to be 1:409,600, 1:819,200, and 1:819,200, respectively (Figure 2B). The isotypes of the three mAbs (3A8, 11E6, and 12D1) were determined to be IgG1, IgG2b, and IgG2a, respectively (Figure S1). WB indicated that the three mAbs could specifically recognize exogenous GP5 protein in HEK293T cells transfected with pCAGGS-GP5-HA (Figure 2C) and endogenous GP5 protein in CD163-3D4/21 cells infected with the NADC30-like PRRSV SD17-38 strain (Figure 2D). In both cases, the detected GP5 proteins had a molecular weight of 25 kD, as expected (Figure 2C,D). The similar results from an immunofluorescence assay (IFA) further confirmed that the three mAbs exhibited strong specificity for both exogenous GP5 (Figure 3) and endogenous GP5 (Figure 4). In both cases, the detected GP5 proteins were located in the cytoplasm (Figure 3 and Figure 4). These results implied that the three mAbs (3A8, 11E6, and 12D1) displayed a specific reaction with PRRSV and its GP5 protein, recognizing linear epitopes of GP5. Moreover, these mAbs could be used for different immune assays (ELISA, WB, and IFA), signifying the application value.

### 2.3. Precise Mapping and Identification of Linear B Cell Epitopes Recognized by GP5 mAbs

**Figure 5:**
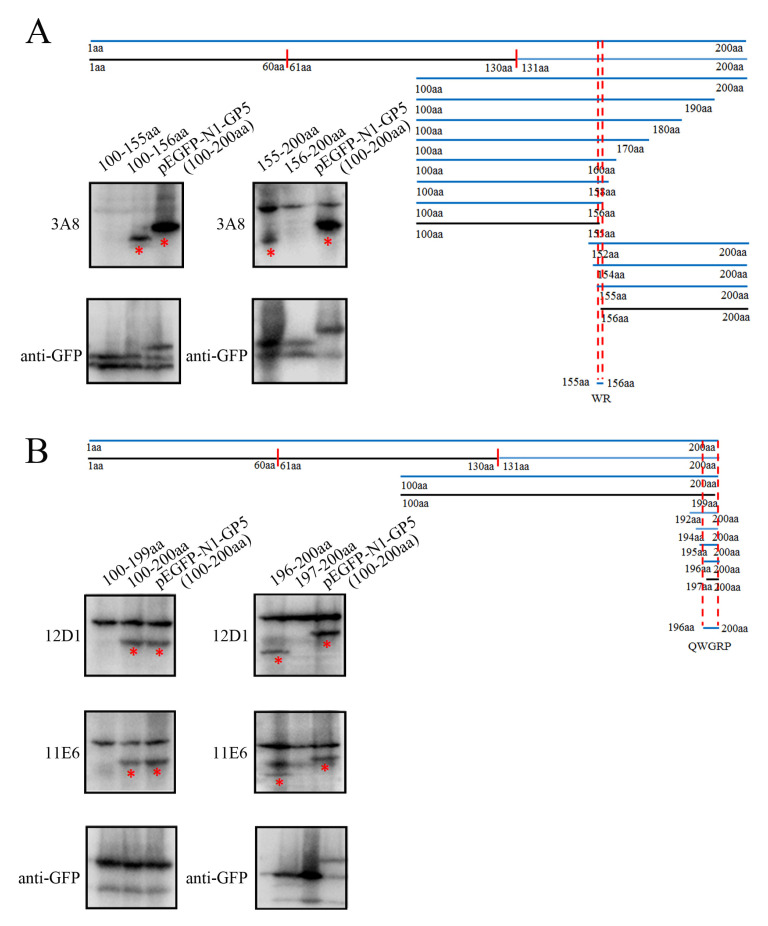
The precise identification of linear B cell epitopes recognized by the GP5 mAbs. (**A**) Schematic diagram of linear B cell epitope precise mapping by the GP5 mAb 3A8. The precise epitope (^155^WR^156^) recognized by the GP5 mAb is shown. WB detected the reactivity of the GP5 mAb 3A8 with the GP5 protein with and without the critical amino acids at both the N- and C-terminal ends. (**B**) Schematic diagram of linear B cell epitope precise mapping by the GP5 mAbs 11E6 and 12D1. The precise epitope (^196^QWGRP^200^) recognized by both of the GP5 mAbs is shown. WB detected the reactivity of the GP5 mAbs 11E6 and 12D1 with the GP5 protein with and without the critical amino acids at both the N- and C-terminal ends. The mAb-reacted GP5 protein bands are marked with red stars underneath.

To identify the precise linear B cell epitopes recognized by the three mAbs, different truncated GP5 fragments were designed and their reactions with three mAbs (3A8, 11E6 and 12D1) were identified by WB (Figure 5). Based on the reactivity of various GP5 fragments with the three GP5 mAbs, we determined the critical amino acids at both the N-terminal and C-terminal ends of the PRRSV GP5 protein and defined the precise antigenic epitopes recognized by the three mAbs (Figure 5 and Figure S2). Taking the above-described results together, the 3A8 mAb recognized minimal linear antigenic epitope ^155^WR^156^ (Figure 5A), whereas the 11E6 and 12D1 mAbs recognized the same minimal linear antigenic peptide ^196^QWGRP^200^ (Figure 5B).

### 2.4. Conservation Analysis of the Identified Antigenic Epitopes Across Different PRRSV Strains

**Figure 6:**
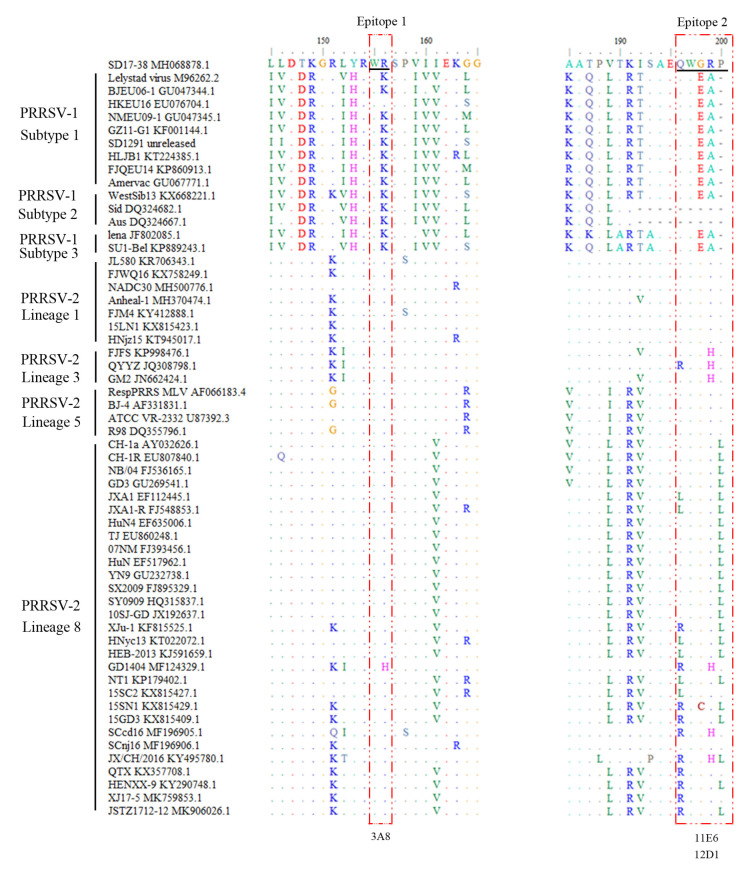
Conservation analysis of the identified antigenic epitopes across different PRRSV strains. The GP5 protein sequences of the PRRSV strains were downloaded from the GenBank database, and BioEdit software (version 7.2) was used for the alignment analysis as referenced to the two identified GP5 epitope sequences. The epitope-containing regions are the boxed areas. “-” indicates identical amino acids to the reference sequence. Colored letters indicate the different amino acids. The corresponding mAbs recognizing the epitopes are marked at the bottom. The alignment of the representative PRRSV strains is shown, including those of PRRSV-1 subtypes 1–3, and PRRSV-2 lineages 1, 3, 5, and 8, with their corresponding names and GenBank accession numbers. The alignment to a complete list of PRRSV GP5 sequences is shown in Table S1.

**Figure 7:**
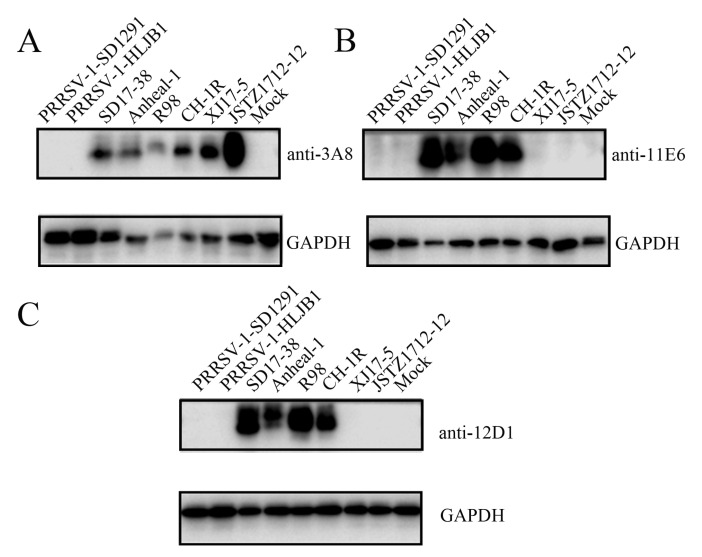
Reactivity of the GP5 specific mAbs with different PRRSV strains by WB. WB was performed to detect the reactivity of GP5 mAbs 3A8 (**A**), 11E6 (**B**), and 12D1 (**C**) with the PRRSV-1 strains (SD1291, HLJB1), NADC30-like PRRSV-2 strain SD17-38, NADC34-like PRRSV-2 strain Anheal-1, Classic PRRSV-2 VR-2332-like strain R98, classical PRRSV-2 vaccine strain CH-1R, and HP-PRRSV-2 virulent strain XJ17-5 and avirulent strain JSTZ1712-12. PRRSV infected cell lysates were used, with mock infected Marc-145 cell lysate as the negative control samples. GAPDH incubated with a mouse mAb (1:1000, Proteintech, Wuhan, China) was used as the internal control.

To perform bioinformatics analysis of the identified GP5 antigenic epitopes from the NADC30-like PRRSV strain, the GP5 protein sequences of different PRRSV strains were downloaded from GenBank and aligned with the two identified GP5 epitope sequences (Figure 6 and Table S1). The results showed that epitope 1, ^155^WR^156^, and epitope 2, ^196^QWGRP^200^, are relatively conserved across different PRRSV strains, with epitope 1 being more conserved than epitope 2 in all the PRRSV-2 strains (Figure 6 and Table S1). Further, the three NADC30-like PRRSV GP5 mAbs were examined by WB for their reactivity with different PRRSV-1 and PRRSV-2 strains, including PRRSV-1 SD1291, PRRSV-1 HLJB1, NADC34-like PRRSV-2 Anheal-1, Classic PRRSV-2 VR-2332-like R98, Classic PRRSV-2 vaccine CH-1R, and HP-PRRSV-2 virulent strain XJ17-5 and avirulent strain JSTZ1712-12 (Figure 7). The 3A8 mAb was verified to specifically react all tested PRRSV-2 strains but neither tested PRRSV-1 strains (Figure 7A). Similarly, IFA showed that the 3A8 mAb reacted with all tested PRRSV-2 strains (Figure S3). The 11E6 and 12D1 mAbs exhibited reactivity towards NADC30-like PRRSV-2 SD17-38, NADC34-like PRRSV-2 Anheal-1, Classic PRRSV-2 VR-2332-like R98, and Classical PRRSV-2 vaccine CH-1R (Figure 7B,C). Together, the results reflected the relative conservation of both epitope 1, ^155^WR^156^, and epitope 2, ^196^QWGRP^200^, across different PRRSV strains.

### 2.5. Neutralizing Activity of Three GP5 mAbs and Development of the Peptide-Based ELISA Detecting PRRSV Antibodies

**Figure 8:**
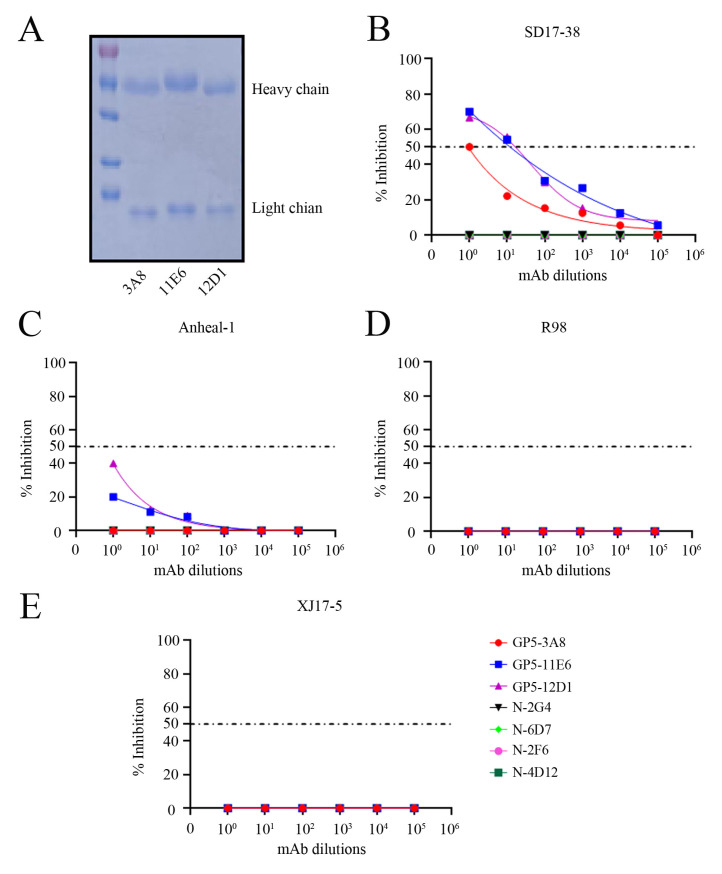
Neutralizing activity of the GP5 mAbs against different PRRSV strains. (**A**) The three GP5 mAbs 3A8, 11E6, and 12D1 were purified and subjected into SDS-PAGE analysis, with the heavy chain and light chain marked. (**B**–**E**) The purified GP5 mAbs, together with N mAbs purified in our lab, were all normalized to 0.1 mg/mL, 10-fold diluted, and incubated with different PRRSV strains, SD17-38 (**B**), Anheal-1 (**C**), R98 (**D**), and XJ17-5 (**E**), followed with infection of 3D4/21-CD163 cells (**B**,**C**) and Marc-145 cells (**D**,**E**) for 72 h. The medium incubated PRRSVs were used as the controls for infection of the 3D4/21-CD163 cells and Marc-145 cells. The infected 3D4/21-CD163 cells were stained with N mAb (6D7) and the goat anti-mouse IgG (H + L) secondary antibody DyLight™ 594 (1:800, Thermo Fisher Scientific) to measure the inhibition of virus infection relative to control infections. The infected Marc-145 cells were observed for CPE to measure the inhibition of the virus infection relative to control infection. The fifty percent end points of neutralization are marked as dotted lines.

We purified the three GP5 ascites mAb IgG as illustrated by the SDS-PAGE analysis (Figure 8A). The purified GP5 mAb IgGs plus PRRSV N mAbs we purified previously [21] were used in virus neutralization test to estimate their neutralizing activity against different PRRSV strains. For NADC30-like PRRSV-2 SD17-38, the GP5 mAbs 11E6 and 12D1 could protect 50% of the cells from viral N protein expression in IFA with dilutions up to 1:148 and 1:165, respectively, whereas 3A8 mAb had the same performance with a dilution only up to 1:10 (Figure 8B). Additionally, neither of the three GP5 mAbs exhibited 50% protection from NADC34-like PRRSV-2 Anheal-1, Classic PRRSV-2 VR-2332-like R98, or HP-PRRSV-2 virulent strain XJ17-5 (Figure 8C–E). As the controls, all four PRRSV N mAbs exhibited no protection against all the tested PRRSV strains (Figure 8B–E). These results suggest that the GP5 mAbs 11E6 and 12D1 have obvious neutralizing activity, whereas GP5 mAb 3A8 has low neutralizing activity against NADC30-like PRRSV.

**Figure 9:**
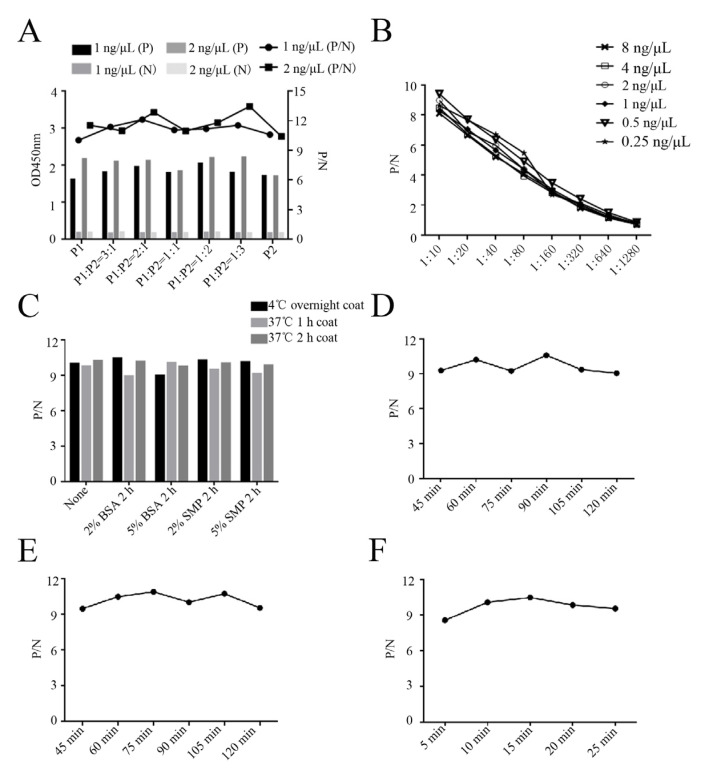
Development and optimization of the peptide-based ELISA. (**A**) Determination of the optimal ratio of the two synthetic peptides P1 (153–158 aa) and P2 (196–200 aa). (**B**) A checkerboard assay determined the optimal working concentration of the synthetic peptides and serum dilution for the peptide-based ELISA. Different amounts of the synthetic peptides (0.25, 0.5, 1, 2, 4, 8 ng/μL) and diluted serum (1:10, 1:20, 1:40, 1:80, 1:160, 1:320, 1:640, 1:1280) were used. (**C**) Determination of the optimal coating and blocking conditions. BSA: bovine serum albumin. SMP: skim milk protein. (**D**–**F**) Determination of the optimal serum incubation time (**D**), secondary antibody incubation time (**E**), and developing time (**F**).

Next, the epitope peptide-based ELISA was developed to detect PRRSV antibodies, including a neutralization antibody. The optimal ratio of the two synthetic peptides P1 (153 to 158 aa) and P2 (196 to 200 aa) was firstly determined to be 1:3, presenting the highest P/N value (Figure 9A). A checkerboard titration assay identified the optimal working concentration of the synthetic peptides as 0.5 ng/μL and the optimal pig serum dilution as 1:10 (Figure 9B). Based on the determined synthetic peptides and serum dilution, the optimal coating of the peptides was at 4 °C overnight and blocking was in 2% BSA buffer for a 2 h incubation (Figure 9C). The optimized incubation times for serum, the secondary antibody, and the substrate were determined to be 90 min (Figure 9D), 75 min (Figure 9E), and 15 min (Figure 9F), respectively.

### 2.6. Specificity, Sensitivity, and Repeatability of the Peptide-Based ELISA

**Figure 10:**
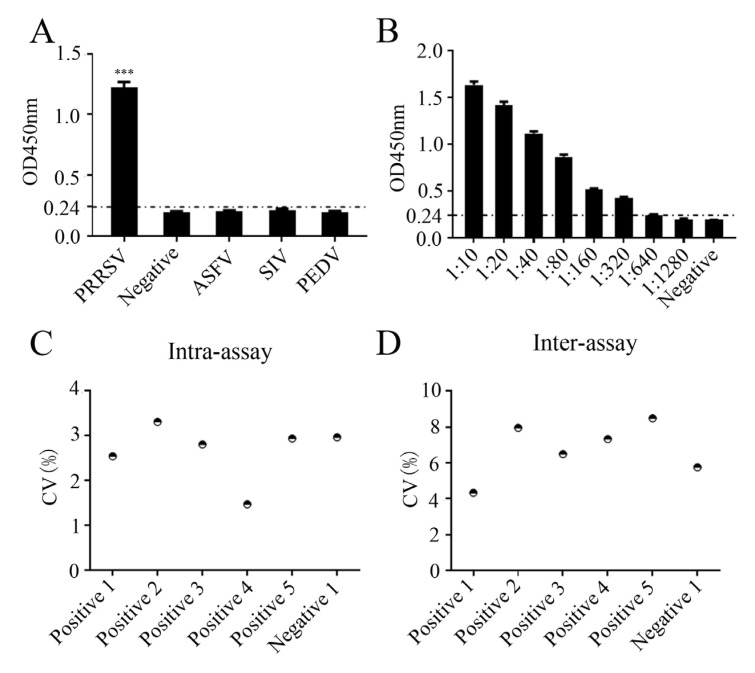
Specificity, sensitivity and repeatability of the peptide-based ELISA. (**A**) Specificity of the peptide-based ELISA was analyzed using PRRSV, ASFV, SIV, and PEDV positive sera, with normal healthy porcine serum as the negative control. (**B**) Sensitivity analysis of the peptide-based ELISA was analyzed using two-fold serially diluted PRRSV-positive pig serum. (**C**,**D**) Intra-assay (**C**) and inter-assay (**D**) repeatability of the peptide-based ELISA (*n* = 3). ***, *p* < 0.001 vs. negative control.

Using 30 negative pig sera, the ELISA cutoff values of the developed peptide-based ELISA were calculated to be 0.227–0.240 based on the optical density 450 nm (OD_450nm_) values of the 30 sera (Table S2). OD_450nm_ values > 0.240 were regarded as positive, whereas OD_450nm_ values < 0.227 were regarded as negative (Table S2). The specificity of the peptide-based ELISA was examined with different positive sera of PRRSV, African swine fever virus (ASFV), swine influenza virus (SIV), and porcine epidemic diarrhea virus (PEDV) (Figure 10A). The peptide-based ELISA reacted only with the PRRSV serum, but not with other sera, demonstrating the specificity of the peptide-based ELISA for PRRSV (Figure 10A). To test the sensitivity of the peptide-based ELISA, PRRSV positive serum was subjected to serial dilutions, followed by detection by the peptide-based ELISA. The maximal dilution of the PRRSV positive serum detectable by peptide-based ELISA was 1:640 (Figure 10B). To evaluate the repeatability of the peptide-based ELISA, the intra- and inter-assay coefficients of variation (CVs) with five positive samples and one negative sample were calculated. As a result, the intra-assay CV was found to range from 1.4 to 3.3% (Figure 10C), while the inter-assay CV ranged from 4.3 to 8.4% (Figure 10D). Both CV values were less than 10%, suggesting that the established peptide-based ELISA for PRRSV antibody detection exhibited excellent repeatability.

### 2.7. Applicability of the Peptide-Based ELISA for Detection of Clinical Samples

To assess applicability of the established peptide-based ELISA, a total of 81 clinical pig serum samples were tested by the peptide-based ELISA. The results showed that of the 81 samples, only 19 were positive tested in the peptide-based ELISA, whereas the other 62 were negative (Table 1). For comparison, the 81 samples were simultaneously detected using the commercial IDEXX PRRSV X3 Ab ELISA. In the IDEXX ELISA, 31 samples were positive and 50 samples were negative (Table 1). Among the 81 samples, 18 were positive by both the peptide-based ELISA and the commercial ELISA, 1 by the peptide-based ELISA only, and 13 by the commercial ELISA only (Table 1 and Table S3). The concordance rate between the peptide-based ELISA and the commercial ELISA was 82.7%, suggesting the applicability of the peptide-based ELISA for detection of clinical samples.

## 3. Discussion

Currently, NADC30-like PRRSV is prevalent and has become the dominant strain in China [5,22,23]. Although the NADC30-like PRRSV is less virulent than the HP-PRRSV, it can still cause abortions in sows and respiratory diseases in fattening pigs [5]. Therefore, it is necessary to investigate the prevention and control of the NADC30-like PRRSV epidemic. GP5, a glycosylated envelope protein, plays a key role in viral virulence and immune response [9,24]. In this study, we developed three NADC30-like PRRSV GP5 specific mAbs, revealed two GP5 antigenic neutralizing epitopes, and established a peptide-based ELISA for detecting NADC30-like PRRSV neutralizing antibodies.

GP5 has been considered as the most relevant antigen for neutralizing antibodies in PRRSV [9,16,25]. Accordingly, the three GP5 mAbs we generated in this study exhibited neutralizing activity; specifically, the mAbs 11E6 and 12D1 possessed obvious neutralizing activity, whereas the mAb 3A8 had low neutralizing activity. The neutralizing activity of these mAbs was only for NADC30-like PRRSV but not for other types of PRRSV strains. It is possible that avidity (total strength of antibody binding to antigen) between the mAbs with different viral antigenic epitopes may be one of the critical factors for the neutralizing ability. In addition, GP5 protein has been found to possess high genetic variation in the PRRSV genome [10,11], suggesting the specific feature of NADC30-like PRRSV and also reflecting the hyper-variability of the GP5 protein.

The PRRSV GP5 N-terminal ectodomain region (1–61 aa) was determined to contain a neutralizing epitope in the middle (37–45 aa) and surrounding immune dominant non-neutralizing epitopes [14,26,27]. Additional studies have identified more antigenic epitopes of GP5, including 1–15 aa, 31–45 aa, 187–200 aa [28], 7–22 aa, 60–66 aa, 76–91 aa, 95–110 aa, 133–140 aa, 154–161 aa, 185–194 aa [29], 152–156 aa, 169–178 aa, and 196–200 aa [30]. In this study, two GP5 precise antigenic epitopes were identified; they are ^155^WR^156^ with the mAb 3A8 and ^196^QWGRP^200^ with both mAbs 11E6 and 12D1. Interestingly, these two GP5 C-terminal epitopes are consistent with the previously identified minimal epitopes 152–156 aa and 196–200 aa [30], demonstrating the relative conservation of the antigenicity in PRRSV-2. Importantly, these two C-terminal GP5 epitopes were shown as the neutralizing epitopes, suggesting multiple neutralization antigenic epitopes exist across the GP5 protein [31,32]. The viral protein antigenic epitopes play an important role in protein structure and antigenic properties [33]. Here, precise identification of the neutralizing epitopes ^155^WR^156^ and ^196^QWGRP^200^ opens new insights on the structure and antigenicity of the NADC30-like PRRSV GP5 protein.

An ELISA is a feasible, sensitive, rapid, and large-scale method for detecting antibodies [15]. Peptide-based ELISAs have been popular due to their low cost, easy preparation, and lack of biosafety concerns [34,35]. Moreover, compared to ELISAs coated with whole antigen or virus, it is targeted at a single or multiple epitopes contained in the synthetic peptide, thus significantly reducing nonspecific reactions [34,35]. In this study, we established a peptide-based ELISA based on the identified GP5 epitopes ^155^WR^156^ and ^196^QWGRP^200^, detecting antibodies including neutralizing antibodies against NADC30-like PRRSV, which displayed high specificity, sensitivity, and repeatability.

In parallel detection of 81 clinical samples, the peptide-based ELISA showed an 82.7% compatibility with the commercial IDEXX ELISA. Among these samples, 18 were positive in both the peptide-based ELISA and the commercial ELISA, whereas 1 was positive in the peptide-based ELISA only and 13 were positive in the commercial ELISA only. It would not be unexpected that significant variation existed between the peptide-based ELISA and commercial ELISA, because both were detecting antibodies to different antigens. For the 1 positive serum in the peptide-based ELISA only, the coated N protein of PRRSV in the IDEXX ELISA kit may present several epitope regions; however, not all of those epitopes may be recognized by PRRSV-positive pig serum antibodies. For the 13 positive in the commercial ELISA only, it is understandable as the commercial ELISA is coated with the most immunogenic and stable N protein of PRRSV, whereas the peptide-based ELISA is coated with synthetic peptides, which are located in the variable GP5 protein.

In conclusion, the three mAbs, 3A8, 11E6, and 12D1, specific to NADC30-like PRRSV GP5 were successfully generated and used for various immune assays. The 11E6 and 12D1 mAbs exhibited obvious neutralizing activity against the NADC30-like PRRSV-2 strain SD17-38, whereas 3A8 mAb showed low neutralizing activity. Two precise linear neutralizing epitopes of GP5 (^155^WR^156^ and ^196^QWGRP^200^) were identified. A peptide-based ELISA was developed using two synthetic GP5 antigenic peptides, and can be used for the detection of neutralizing antibodies against NADC30-like PRRSV, which will contribute to the diagnosis, prevention, and control of the pig disease caused by NADC30-like PRRSV.

## 4. Materials and Methods

### 4.1. Cells, Viruses, and Mice

The 3D4/21-CD163 cells [36] were grown in Roswell Park Memorial Institute 1640 (RPMI-1640; Hyclone Laboratories, Logan, UT, USA) and supplemented with 10% fetal bovine serum (FBS, Eallbio, Beijing, China), whereas the myeloma cells SP2/0, Marc-145, and HEK293T cells were cultured in Dulbecco’s modified Eagle’s medium (DMEM; Life Technologies Corp., Grand Island, NY, USA), containing 10% FBS at 37 °C in 5% CO_2_. Primary porcine alveolar macrophages (PAMs) were prepared using regular bronchoalveolar lavage from 2-month-old domestic pigs, and cultured in RPMI-1640 medium supplemented with 10% FBS.

The PRRSV strains used in this study were all stored in our laboratory, including NADC30-like PRRSV-2 strain SD17-38 (lineage 1, GenBank: MH068878.1) [37], NADC34-like PRRSV-2 strain Anheal-1 (lineage 1, GenBank: MH370474.1, a courtesy from Dr. Xizhao Chen at Anheal Laboratories Co., Ltd. Beijing, China), Classic PRRSV-2 VR-2332-like strain R98 (lineage 5, GenBank: DQ355796.1), classical PRRSV-2 vaccine strain CH-1R (lineage 8, GenBank: EU807840.1), HP-PRRSV-2 virulent strain XJ17-5 (lineage 8, GenBank: MK759853.1) and avirulent strain JSTZ1712-12 (lineage 8, GenBank: MK906026.1) [38], PRRSV-1 strain SD1291 [39], and PRRSV-1 strain HLJB1 (GenBank: KT224385.1) [40]. The 3D4/21-CD163 cells were infected with SD17-38 (0.1 MOI) or Anheal-1 (0.1 MOI) for 72 h; the Marc-145 cells were infected with R98 (0.1 MOI), CH-1R (0.1 MOI), XJ17-5 (0.1 MOI), or JSTZ1712-12 (0.1 MOI) for 72 h; and the primary PAMs were infected with HLJB1 (0.1 MOI) or SD1291 (0.1 MOI) for 72 h.

BALB/c mice were purchased from Yangzhou University animal facility, and this study was approved by the Guide for the Care and Use of Laboratory Animals and Yangzhou University (SYXK(JS)-2022-0044).

### 4.2. Bioinformatics Analysis of the GP5 Protein and Construction of the Recombinant Plasmids

The NADC30-like PRRSV-2 SD17-38 strain (GenBank: MH068878.1) GP5 protein was analyzed for prediction of transmembrane (TM) regions by the online software TMHMM-2.0 (https://services.healthtech.dtu.dk/services/TMHMM-2.0/, accessed on 27 February 2025). The hydrophobicity and hydrophilicity scales of GP5 were analyzed through the online software ProtScale (https://web.expasy.org/protscale/, accessed on 27 February 2025). The B cell antigenic epitopes in the GP5 protein were predicted using an online tool (http://tools.iedb.org/main/bcell/, accessed on 27 February 2025). The signal peptide in the GP5 protein was predicted via an online tool (https://services.healthtech.dtu.dk/services/SignalP-5.0/, accessed on 27 February 2025).

Based on the ORF5 (GP5) sequence of the NADC30-like PRRSV-2 SD17-38 strain and bioinformatics analysis, two pairs of specific PCR primers were designed to amplify two GP5 intramembrane regions (33–65, 126–200 aa) separately. These two overlapping PCR products were cloned into the *Sal*I/*EcoR*V sites of the Gateway entry vector pENTER4-MCS-2HA using the ClonExpress Ultra One Step Cloning Kit (Vazyme, Nanjing, China). By LR recombination (Gateway LR Clonase™ II Enzyme mix, ThermoFisher Scientific, Shanghai, China), the ORF5 (GP5) fused truncation fragment was transferred from the pENTER4-2HA vector to the Destination vector pDEST527 (Addgene, Watertown, MA, USA) to obtain the recombinant prokaryotic pDEST527-GP5-2HA. The eukaryotic plasmid expressing the HA-tagged full length GP5 was made by cloning the ORF5 (GP5) sequence into the *EcoR*I/*EcoR*V sites of vector pCAGGS-2HA using seamless cloning (2 × MultiF Seamless Assembly Mix, Abclonal, Wuhan, China). The eukaryotic plasmids expressing GFP-tagged GP5 and its truncation derivatives were made by cloning the corresponding gene fragments into the *Xhol*I/*EcoR*I sites of the eukaryotic vector pEGFP-N1 using seamless cloning. The cloning PCR primers used for the study are all listed in Table S4. All recombinant plasmids were confirmed by Sanger DNA sequencing.

### 4.3. Expression and Purification of the Recombinant GP5 Truncation Protein

The recombinant pDEST527-GP5-2HA was transformed into *E. coli* BL21 (DE3) competent cells. After induction in 1 mM isopropyl β-D-1-thiogalactopyranoside (IPTG), the recombinant GP5 protein was expressed and then subjected to purification. The expressed GP5 protein in the form of an inclusion body, was solubilized by 8 M urea solution, renatured by a stepwise dialysis (6 M, 4 M, 2 M, 1 M urea), and finally dissolved in phosphate buffered saline (PBS). The identification of both expressed and purified GP5 proteins was performed by SDS-PAGE with Coomassie brilliant blue staining. The expressed GP5 protein was also detected using Western blotting with the His mAb and HA mAb (each 1:1000, TransGen Biotech, Beijing, China).

### 4.4. Immunization of the Mice and Monoclonal Antibody (mAb) Production and Purification

The 6-week-old BALB/c mice were received and an initial immunization with 100 μg of purified GP5 protein emulsified 10:1 with Montanide gel (SEPPIC SA, Cedex, France) was conducted by subcutaneous injection on the back. Two weeks later, 50 μg of purified GP5 protein was administered via intrasplenic injection to booster immunization. One week later, the spleen cells from the immunized mice were harvested to fuse with myeloma cells SP2/0 using PEG1500 following a routine procedure. The hybridoma supernatants were collected for GP5 antibody detection by GP5 coated indirect ELISA. The hybridoma cells that tested positive were subcloned three times by limiting dilution. The supernatants from these hybridoma cells were further confirmed by Western blotting and indirect an immunofluorescence assay for secreting a GP5 specific mAb. Ascites containing GP5 mAbs were collected from the mice with intraperitoneal injection of sterile liquid paraffin and subsequent hybridoma cells. The GP5 mAbs of the ascites were purified by the Protein A/G Agarose column (Santa Cruz Biotechnology, Dallas, TX, USA).

### 4.5. GP5 Indirect Enzyme-Linked Immunosorbent Assay (ELISA)

The indirect ELISA was used to screen hybridoma cell clones secreting antibodies against the GP5 protein or to measure the titration of the ascites mAbs. Briefly, 96-well plates were coated with 0.625 μg/mL recombinant GP5 truncation protein (100 μL/well) overnight at 4 °C. The plates were washed three times with PBST (1 × phosphate-buffered saline containing 0.05% Tween-20) and blocked with 5% skim milk for 2 h. Then, hybridoma culture supernatants (100 μL/well) were added to each well and incubated for 1 h at 37 °C. After washing three times, horseradish peroxidase (HRP)-conjugated goat anti-mouse IgG (1:10,000 dilution, 100 μL/well, TransGen Biotech, Beijing, China) was added and incubated for 1 h at 37 °C. The substrate 3,3′,5,5′-tetramethylbenzidine (TMB) (50 μL/well, Beyotime, Beijing, China) was added and incubated for 15 min in the dark. Finally, 2M H_2_SO_4_ (50 μL/well) was added to terminate the reaction, followed by reading of the absorbance at 450 nm (OD_450_) with a spectrophotometer (ALL-SHENG, Hangzhou, China). The ratios of hybridoma culture supernatants to negative SP2/0 supernatant (P/N) were calculated, with P/N ≥ 2.1 as positive.

### 4.6. Western Blotting (WB)

Cells were lysed in radio-immunoprecipitation assay (RIPA) buffer and protein samples were harvested by centrifugation. The protein samples were subjected to sodium dodecyl sulfate-polyacrylamide gel electrophoresis (SDS-PAGE) and transferred to polyvinylidene difluoride (PVDF) membranes. After blocking with 5% skim milk solution at room temperature (RT) for 1 h, membranes were incubated with the appropriate primary antibodies (1:1000) at 4 °C overnight and then with secondary antibody HRP-conjugated goat anti-mouse IgG (1:10,000, TransGen Biotech, Beijing, China) at 37 °C for 1 h. Finally, the membrane was exposed with an enhanced chemiluminescence (ECL) substrate (Tanon, Shanghai, China). Images were taken by an imaging system (Tanon, Shanghai, China).

### 4.7. Immunofluorescence Assay (IFA)

3D4/21-CD163 cells seeded in 12-well plates were infected with SD17-38 strain at 0.1 multiplicity of infection (MOI) for 72 h, whereas HEK293T cells grown in 12-well plates were transfected with eukaryotic pGFP-N1-GP5 plasmid for 24 h. Cells were washed twice with PBS, fixed with 4% paraformaldehyde (Beyotime Biotech, Shanghai, China) for 10 min, and permeabilized with 0.1% Triton X-100 for 10 min at RT. After washing, cells were blocked with 1% bovine serum albumin (BSA) at 37 °C for 1 h and incubated with the GP5 mAbs (1:200) at 4 °C overnight. After three PBS washes, the goat anti-mouse IgG (H + L) secondary antibody DyLight™ 594 (1:800, Thermo Fisher Scientific, Sunnyvale, CA, USA) was added to continue incubation away from light for 1 h. Cell nuclei were stained with 4’, 6-diamidino-2-phenylindole (DAPI, ThermoFisher Scientific) for 10 min in the dark. Signals were observed under a fluorescence microscope (Leica, SPE, Buffalo Grove, IL, USA).

### 4.8. Precise Identification of the mAb Recognizing Epitopes and the Epitope Conservation Analysis

To identify the B-cell epitope, the GP5 protein was first divided into three overlapping fragments. The reactivity of the overlapping fragments with GP5 mAbs was examined by WB. Subsequently, the reacted fragment was subjected to fine progressive truncation at both the N- and C-terminal ends, and the reactivity of these truncated fragments with GP5 mAbs was examined by WB. As such, the critical amino acids at both the N- and C-terminal ends were identified for reactivity with GP5 mAbs. The precise epitope sequences could be deduced and defined based on the critical amino acids at both ends. All the truncated GP5 fragments were ligated into the vector pEGFP-N1 using seamless cloning and fused with a GFP-tag. The recombinant vectors were transfected into HEK293T cells for expression of different truncated fragments. The fragment reacting with both GP5 mAb and anti-GFP mAb (TransGen Biotech) was determined as the epitope containing region.

To assess the conservation of epitopes recognized by the GP5 mAb among different PRRSV-1 and PRRSV-2 strains, the GP5 protein sequences of all PRRSV-1 and PRRSV-2 were downloaded from GenBank and aligned with the mAb recognizing epitope using the BioEdit software (version 7.2). The peptides (P1, ^153^YRWRSP^158^; P2, ^196^QWGRP^200^) were synthesized by Sangon Biotech Co., Ltd. (Shanghai, China).

### 4.9. Virus Neutralization Test by the mAbs

The GP5 mAbs (1 mg/mL) and N mAb were purified in our lab were all normalized to 0.1 mg/mL before being used for virus neutralization test. The 10-fold serial diluted purified GP5 mAbs were mixed with an equal volume of 200 TCID_50_/0.1 mL of virus for 1 h at 37 °C in 5% CO_2_. Then, the mAb-treated and PBS-treated viruses were added to a 96-well plate containing 3D4/21-CD163 or Marc-145 cells. At 2 h post infection, the media for the cells were changed and the cells were further incubated for 70 h. The inhibition of virus infections was calculated by counting the IFA positive spots in SD17-38 and Anheal-1 infected 3D4/21-CD163 cells or the PRRSV-specific cytopathic effect (CPE) in R98 and XJ17-5 infected Marc-145 cells. The fifty percent end points of the neutralization titers were calculated and applied [25].

### 4.10. Development and Optimization of GP5 Peptide-Based ELISA

The ELISA plate wells were coated with 100 μL synthetic GP5 antigenic peptides (0.25, 0.5, 1, 2, 4, 8 ng/μL) in 0.05 M carbonate and bicarbonate buffer (pH 9.6) overnight at 4 °C. After washing with PBST, the wells were blocked with 5% skim milk for 2 h and incubated with the diluted PRRSV-positive pig serum samples (1:10–1:1280, 100 μL/well) for 1 h at 37 °C. Normal healthy porcine serum was used as the negative control. The secondary antibody, HRP-conjugated goat anti-swine IgG (1:10,000, Proteintech, Wuhan, China), was added at 100 μL/well and incubated at 37 °C for 1 h. Then, 50 μL of 3,3′,5,5′-tetramethylbenzidine (TMB) was added into each well and incubated for 15 min in the dark, followed by adding 50 μL of 2 M H_2_SO_4_ per well to terminate the reaction. The optical density (OD) value was determined at 450 nm by a spectrophotometer (ALL-SHENG, Hangzhou, China).

To achieve good peptide-based ELISA performance, various experimental conditions were optimized, including the coating conditions of synthetic peptide, blocking conditions, dilution of the serum, serum incubation time, secondary antibody incubation time, and developing time. The optimal conditions were determined while the largest ratios of OD_450_ values of the positive sera relative to negative serum (P/N) were achieved.

### 4.11. Cut-Off Values, Specificity, and Sensitivity of the Peptide-Based ELISA

The mean of the OD_450_ values and standard deviations (SD) of thirty PRRSV-negative pig serum samples were calculated, and the cut-off values of the peptide-based ELISA were determined to be between the mean + 2SD and mean + 3SD. The specificity of the peptide-based ELISA was evaluated using positive pig serum samples, including PRRSV, African swine fever virus (ASFV), swine influenza virus (SIV), and porcine epidemic diarrhea virus (PEDV). The sensitivity of the peptide-based ELISA was determined by testing 2-fold serial dilutions of PRRSV-positive pig serum. All the pig serum samples were stored in our laboratory.

### 4.12. Repeatability and Application of the Peptide-Based ELISA

The repeatability of the peptide-based ELISA was assessed using five PRRSV positive serum samples and one negative serum sample. To test intra-assay repeatability, three replicates of each sample were examined using the same batch of pre-coated plates. To test the inter-assay repeatability, three batches of pre-coated ELISA plates were used to detect each sample. Based on three replications of each test, the coefficient of variation (CV) was calculated as CV = (SD/Mean) × 100%. The intra- or inter-assay repeatability of the peptide-based ELISA was evaluated according to the CV.

The 81 clinical serum samples from PRRSV-negative pigs and PRRSV-positive pigs were tested by the developed peptide-based ELISA and IDEXX PRRSV X3 Ab ELISA (IDEXX, Westbrook, ME, USA). The overall agreement between the peptide-based ELISA and IDEXX PRRSV X3 Ab ELISA was evaluated.

### 4.13. Statistical Analysis

The data are expressed as means ± SD (*n* = 3). Results were analyzed by GraphPad Prism 8.0 software (San Diego, CA, USA). Statistical analyses were performed by Student *t* test. A *p* value less than 0.05 was considered statistically significant.

## Figures and Tables

**Table 1:** Detection of the serum samples using the peptide-based ELISA together with the IDEXX PRRSV X3 Ab ELISA.

Samples	Peptide-Based ELISA		IDEXX		Coincidence Rate (%)
	Positive	Negative	Positive	Negative	
Sera (81)	19 (1 *)	62 (13 *)	31 (13 ^#^)	50 (1 ^#^)	82.7

Note: * denotes the numbers of positive or negative results by only the peptide-based ELISA, whereas # denotes the numbers of positive or negative results by only the IDEXX PRRSV X3 Ab ELISA.

## Data Availability

The authors confirm that the data supporting the findings of this study are available within the article and its Supplementary Materials.

## Supplementary Materials

The following supporting information can be downloaded at: https://www.mdpi.com/article/10.3390/ijms26062619/s1.
