# Supplementary material for: A Novel Peptide-Based Enzyme-Linked Immunosorbent Assay (ELISA) for Detection of Neutralizing Antibodies Against NADC30-like PRRSV GP5 Protein
Source: Int J Mol Sci. 2025 Mar 14;26(6):2619. doi: 10.3390/ijms26062619 (PMC11941917; doi:10.3390/ijms26062619)
Supplement: Supplementary file 1 [file ijms-26-02619-s001.zip › Supplementary Figures.pdf]

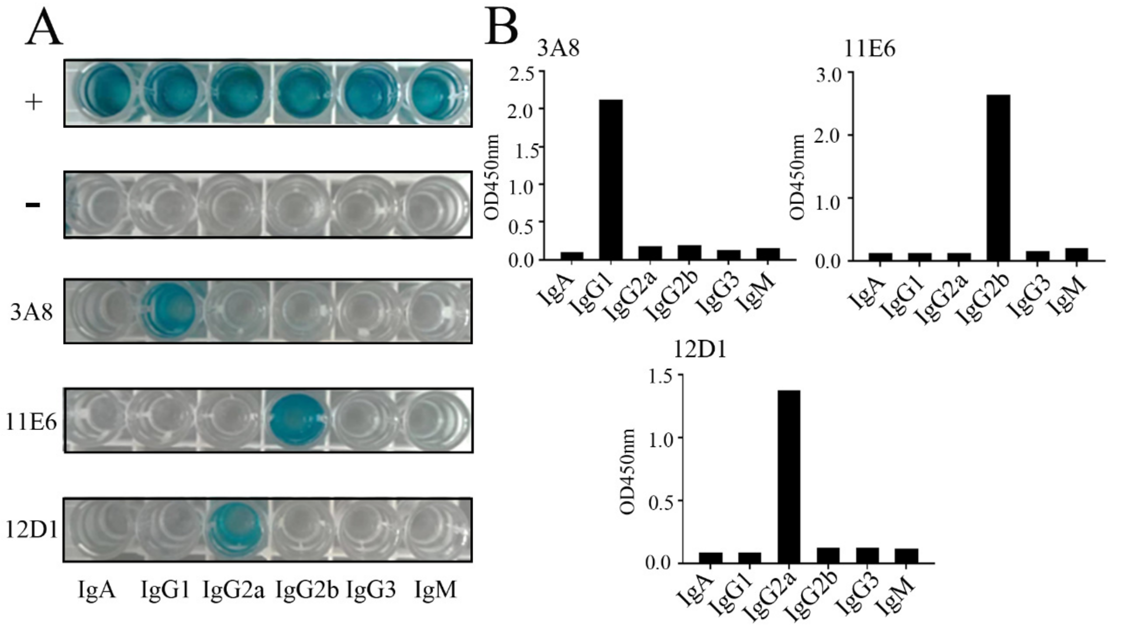

Fig S1. Isotype identification of GP5-specific mAbs

The commercial mouse monoclonal antibody isotype identification kit (CELLWAY-LAB, Luoyang China) was used to perform isotype determination of mAbs 3A8, 11E6, and 12D1. Briefly, 50 µL each mAb solutions mixed with 50 µL sample diluent from the kit was dispensed to the detection plate wells, with each mAb 6 replicate wells (100 µL/well). In parallel, the positive and negative solutions from the kit were also dispensed, with each 6 replicates. After incubating at 37°C for 30 min, the plate was washed 5 times and the 6 types of enzyme-linked secondary antibodies were added into the wells of each samples, accordingly (100 µL/well). After incubating at 37°C for 30 min, the plate was washed, and the substrates A and B from the kit were added (50 µL each) for the chromogenic reaction at 37°C for 20 min in the dark. The reaction was stopped by the stopping solution (50 µL/well) and the optical density at 450 nm (OD450) was measured by a spectrophotometer (ALL-SHENG, Hangzhou, China). The mAb isotype was determined based on the higher OD450 value of the exact type of 6 secondary antibodies. The color reaction image was taken (**A**) and the OD450 values was shown (**B**). Based on the reaction results, the isotypes of GP5 mAbs 3A8, 11E6, and 12D1 were IgG1, IgG2b, and IgG2a, respectively.

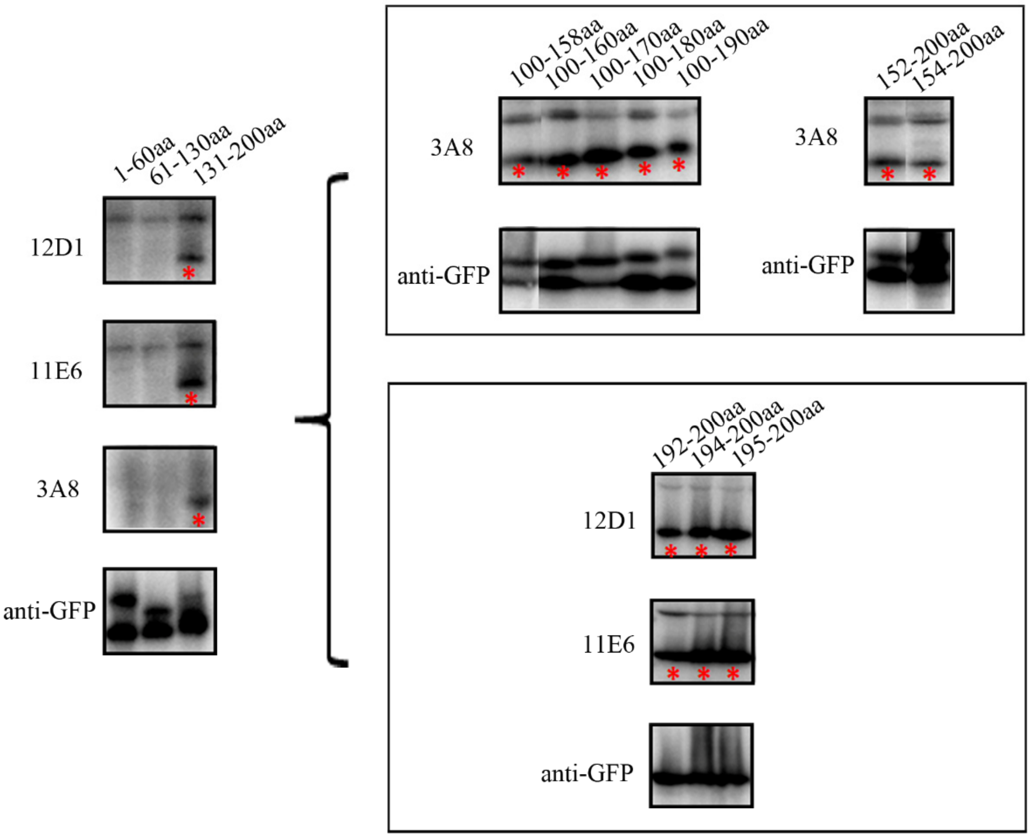

Fig S2. Precise identification of the linear B cell epitopes recognized by the GP5 mAbs. The reactions of mAbs 3A8, 11E6, and 12D1 with the indicated GP5 truncated proteins beyond the **Fig 5** were determined by Western blotting. The mAb reacted GP5 protein bands were marked with red stars underneath.

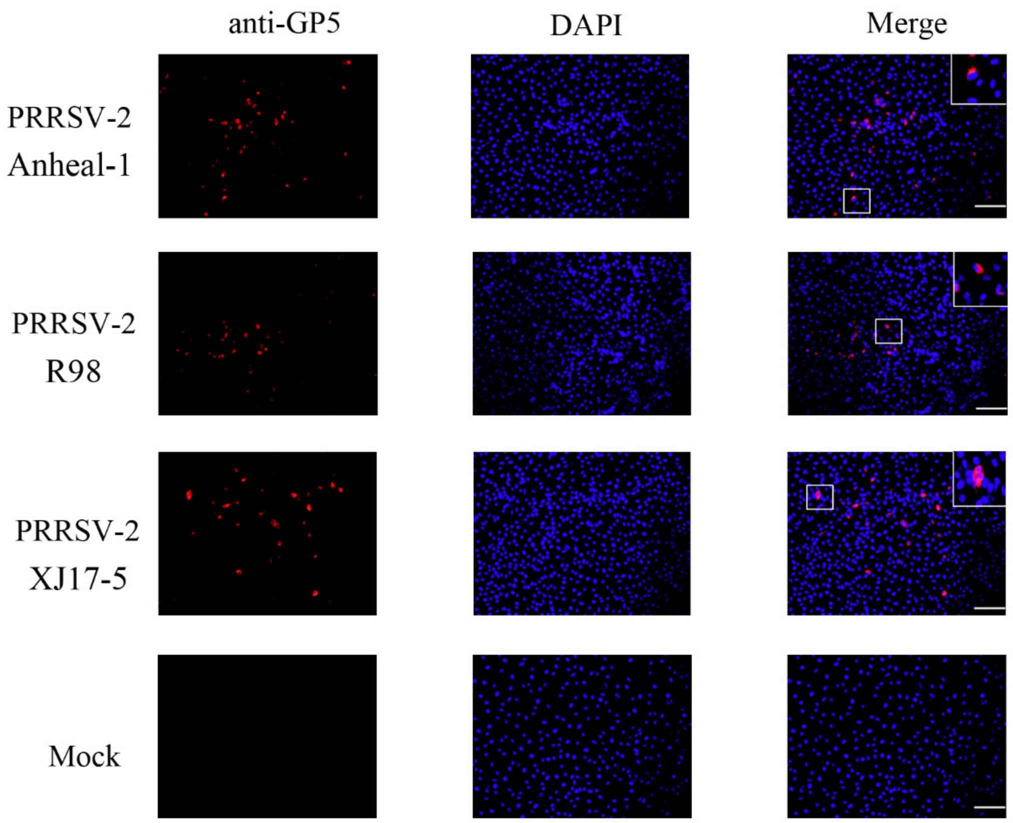

Fig S3. Reactivity of GP5 specific mAb 3A8 with different PRRSV-2 strains in IFA

3D4/21-CD163 cells were infected with Anheal-1 (0.1 MOI), R98 (1 MOI), and XJ17-5 (1 MOI) for 72 h. Mock infected 3D4/21-CD163 cells were used as control. The cells were fixed, stained with GP5 mAb 3A8, and incubated with anti-mouse IgG (H+L) secondary antibody DyLight™ 594 (red). Cell nuclei were counter stained with DAPI (blue). The boxed areas are magnified and placed on the upper-right corners of the merged images, clearly illustrating the cytoplasmic localization of the GP5 protein. Scar bar, 100 μm.
