# Supplementary material for: A Novel Peptide-Based Enzyme-Linked Immunosorbent Assay (ELISA) for Detection of Neutralizing Antibodies Against NADC30-like PRRSV GP5 Protein
Source: Int J Mol Sci. 2025 Mar 14;26(6):2619. doi: 10.3390/ijms26062619 (PMC11941917; doi:10.3390/ijms26062619)
Supplement: Supplementary file 1 [file ijms-26-02619-s001.zip › Supplementary Table 2.pdf]

Table S2. Negative sample OD_{450} values in peptide-based ELISA detection

No. of sample	OD450 nm value	No. of sample	OD450 nm value
1	0.193	16	0.198
2	0.214	17	0.220
3	0.194	18	0.225
4	0.197	19	0.211
5	0.208	20	0.195
6	0.185	21	0.184
7	0.196	22	0.222
8	0.186	23	0.218
9	0.193	24	0.192
10	0.217	25	0.191
11	0.195	26	0.221
12	0.220	27	0.189
13	0.187	28	0.211
14	0.199	29	0.199
15	0.193	30	0.188
