# Supplementary material for: A Novel Peptide-Based Enzyme-Linked Immunosorbent Assay (ELISA) for Detection of Neutralizing Antibodies Against NADC30-like PRRSV GP5 Protein
Source: Int J Mol Sci. 2025 Mar 14;26(6):2619. doi: 10.3390/ijms26062619 (PMC11941917; doi:10.3390/ijms26062619)
Supplement: Supplementary file 1 [file ijms-26-02619-s001.zip › Supplementary Table 3.pdf]

Table S3. Detection of clinical samples using the peptide-based ELISA relative to the IDEXX PRRSV X3 Ab ELISA

Number	Samples	Regions	Peptide-based ELISA	IDEXX
1	Serum	Jiangsu	-	-
2	Serum	Jiangsu	-	-
3	Serum	Jiangsu	-	-
4	Serum	Jiangsu	-	-
5	Serum	Jiangsu	-	-
6	Serum	Jiangsu	-	-
7	Serum	Jiangsu	-	-
8	Serum	Jiangsu	-	-
9	Serum	Jiangsu	-	-
10	Serum	Jiangsu	-	-
11	Serum	Jiangsu	-	-
12	Serum	Jiangsu	-	-
13	Serum	Jiangsu	+	+
14	Serum	Jiangsu	+	+
15	Serum	Jiangsu	+	+
16	Serum	Jiangsu	+	+
17	Serum	Jiangsu	-	+
18	Serum	Jiangsu	-	+
19	Serum	Jiangsu	-	+
20	Serum	Jiangsu	-	-
21	Serum	Jiangsu	-	-
22	Serum	Jiangsu	-	+
23	Serum	Jiangsu	+	+
24	Serum	Jiangsu	+	+
25	Serum	Jiangsu	-	-
26	Serum	Jiangsu	-	-
27	Serum	Jiangsu	-	-
28	Serum	Jiangsu	-	-
29	Serum	Jiangsu	-	-
30	Serum	Jiangsu	-	-
31	Serum	Jiangsu	-	-
32	Serum	Jiangsu	-	-
33	Serum	Jiangsu	-	-

34	Serum	Jiangsu	-	-
35	Serum	Jiangsu	-	-
36	Serum	Jiangsu	-	-
37	Serum	Jiangsu	-	+
38	Serum	Jiangsu	-	-
39	Serum	Jiangsu	-	-
40	Serum	Jiangsu	+	+
41	Serum	Jiangsu	-	+
42	Serum	Jiangsu	+	+
43	Serum	Jiangsu	+	+
44	Serum	Jiangsu	+	+
45	Serum	Jiangsu	-	+
46	Serum	Jiangsu	+	-
47	Serum	Jiangsu	+	+
48	Serum	Jiangsu	+	+
49	Serum	Jiangsu	+	+
50	Serum	Jiangsu	+	+
51	Serum	Jiangsu	+	+
52	Serum	Jiangsu	-	-
53	Serum	Jiangsu	-	-
54	Serum	Jiangsu	-	-
55	Serum	Jiangsu	-	-
56	Serum	Jiangsu	-	-
57	Serum	Jiangsu	-	-
58	Serum	Jiangsu	-	-
59	Serum	Jiangsu	-	-
60	Serum	Jiangsu	-	-
61	Serum	Jiangsu	+	+
62	Serum	Jiangsu	-	+
63	Serum	Jiangsu	-	+
64	Serum	Jiangsu	-	+
65	Serum	Jiangsu	-	+
66	Serum	Jiangsu	-	-
67	Serum	Jiangsu	-	-
68	Serum	Jiangsu	-	+
69	Serum	Jiangsu	+	+
70	Serum	Jiangsu	-	+

71	Serum	Jiangsu	+	+
72	Serum	Jiangsu	-	-
73	Serum	Jiangsu	-	-
74	Serum	Jiangsu	-	-
75	Serum	Jiangsu	-	-
76	Serum	Jiangsu	-	-
77	Serum	Jiangsu	-	-
78	Serum	Jiangsu	-	-
79	Serum	Jiangsu	-	-
80	Serum	Jiangsu	-	-
81	Serum	Jiangsu	-	-

Note: The positive samples were marked differently, with both positive shaded with grey; peptide-based ELISA only positive colored red and shaded yellow, and IDEXX PRRSV X3 Ab ELISA only positive colored red.
