# Supplementary material for: A Novel Peptide-Based Enzyme-Linked Immunosorbent Assay (ELISA) for Detection of Neutralizing Antibodies Against NADC30-like PRRSV GP5 Protein
Source: Int J Mol Sci. 2025 Mar 14;26(6):2619. doi: 10.3390/ijms26062619 (PMC11941917; doi:10.3390/ijms26062619)
Supplement: Supplementary file 1 [file ijms-26-02619-s001.zip › Supplementary Table 4.pdf]

Table S4. The PCR primers used in this study

Name	Sequences (5' - 3')
pENTER4-GP5-2HA-1	F: ACCATGGGAACCAATTCAGTCGACAACAGCAGCTCCCATTTAC<br>R: GGACATGCAATTTTTCGCCAACCTCTCCACTGCCCAGTCAAAAT
pENTER4-GP5-2HA-2	F: AAAATTTTGACTGGGCAGTGGAGAGGTTGGCGAAAAATTGCATG<br>R: ATCGTATGGGTAGCTGGTGATATCTGGACGACCCCATTGTTC
pCAGGS-GP5-2HA	F: CATTTTGGCAAAgaattc**ATG**ATGTTGGGGAAATGCTTG<br>R: TATGGGTAGCTGGTgatatcTGGACGACCCCATTGTTC
pEGFP-N1-GP5	F: ACTCAGATctcgag**ATG**ATGTTGGGGAAATGCTTG<br>R: CGTCGACTGCAgaattcCTGGACGACCCCATTGTTC
pEGFP-N1-1-60aa	F: ACTCAGATctcgag**ATG**ATGTTGGGGAAATGCTTG<br>R: CGTCGACTGCAgaattcCAAAATTTTGTTTTTCAGCCAATCAG
pEGFP-N1-61-130aa	F: ACTCAGATctcgag**ATG**GACTGGGCAGTGGAGAC<br>R: CGTCGACTGCAgaattcCATTTTTCGCCAACCTAATGGC
pEGFP-N1-131-200aa	F: ACTCAGATctcgag**ATG**TGCATGTCCTGGCGCT<br>R: CGTCGACTGCAgaattcCTGGACGACCCCATTGTTC
pEGFP-N1-100-200aa	F: GACTCAGATctcgag**ATG**GGTTATTATCATGGGCG<br>R: CGTCGACTGCAGAATTCCTGGACGACCCCATTGTTC
pEGFP-N1-100-190aa	F: GACTCAGATctcgagA**TG**GGTTATTATCATGGGCG<br>R: CGACTGCAgaattcCGGTTACAGGGGTTGCCGCGGA
pEGFP-N1-100-180aa	F: GACTCAGATctcgag**ATG**GGTTATTATCATGGGCG<br>R: CGTCGACTGCAgaattcCCACAACTCTCTTGAGGTC
pEGFP-N1-100-170aa	F: GACTCAGATctcgag**ATG**GGTTATTATCATGGGCG<br>R: GTCGACTGCAgaattcCCCCAACATCAACTTTACC
pEGFP-N1-100-160aa	F: GACTCAGATctcgag**ATG**GGTTATTATCATGGGCG<br>R: CGACTGCAGAATTCCGATGACAGGTGACCGCCAG
pEGFP-N1-100-158aa	F: GACTCAGATctcgag**ATG**GGTTATTATCATGGGCG<br>R: GTCGACTGCAgaattcCAGGTGACCGCCAGCG
pEGFP-N1-100-156aa	F: GACTCAGATctcgag**ATG**GGTTATTATCATGGGCG<br>R: CGACTGCAgaattcCCCGCCAGCGGTAGAGTC
pEGFP-N1-100-155aa	F: GACTCAGATctcgag**ATG**GGTTATTATCATGGGCG<br>R: TCGACTGCAgaattcCCCAGCGGTAGAGTCTGC
pEGFP-N1-152-200aa	F: GACTCAGATctcgag**ATG**CTCTACCGCTGGCGGTCA<br>R: CGTCGACTGCAgaattcCTGGACGACCCCATTGTTC
pEGFP-N1-154-200aa	F: GACTCAGATctcgag**ATG**CGCTGGCGGTCACCT<br>R: CGTCGACTGCAgaattcCTGGACGACCCCATTGTTC
pEGFP-N1-155-200aa	F: ACTCAGATctcgag**ATG**TGGCGGTCACCTGTCA<br>R: CGTCGACTGCAgaattcCTGGACGACCCCATTGTTC
pEGFP-N1-156-200aa	F: ACTCAGATctcgag**ATG**CGGTCACCTGTCATCA<br>R: CGTCGACTGCAgaattcCTGGACGACCCCATTGTTC
pEGFP-N1-100-199aa	F: GACTCAGATctcgag**ATG**GGTTATTATCATGGGCG<br>R: TCGACTGCAgaattcCACGACCCCATTGTTCCG
pEGFP-N1-192-200aa	F: AGATctcgag**ATG**ATTTCAGCGGAACAATGGGGTCGTCCAGGAATTCTGCAGTC<br>R: GACTGCAgaattcCTGGACGACCCCATTGTTCCGCTGAAATCATCTCGAGATCT

pEGFP-N1-194-200aa	F: CAGATctcgag**ATG**GCGGAACAATGGGGTCGTCCAGGAATTCTGCAGT
	R: ACTGCAgaattcCTGGACGACCCCATTGTTCCGCCATCTCGAGATCTG
pEGFP-N1-195-200aa	F: ACTCAGATctcgag**ATG**GAACAATGGGGTCGTCCAGGAATTCTGCAGTCGACGGT
	R: ACCGTCGACTGCAgaattcCTGGACGACCCCATTGTTCCATCTCGAGATCTGAGTC
pEGFP-N1-196-200aa	F: CAGATctcgag**ATG**CAATGGGGTCGTCCAGGAATTCTGCAGTCG
	R: CGACTGCAgaattcCTGGACGACCCCATTGCATCTCGAGATCTG
pEGFP-N1-197-200aa	F: CTCAGATctcgag**ATG**TGGGGTCGTCCAGGAATTCTGCAGTC
	R: GACTGCAgaattcCTGGACGACCCCACATCTCGAGATCTGAG
